# A new metabolic path in type 3 rickets

**DOI:** 10.1111/febs.70382

**Published:** 2025-12-30

**Authors:** Toshiya Senda, Yoshihisa Hirota

**Affiliations:** ^1^ Structural Biology Research Center, Institute of Materials Structure Science High Energy Accelerator Research Organization (KEK) Tsukuba Japan; ^2^ Laboratory of Biochemistry, Department of Bioscience and Engineering, College of Systems Engineering and Science Shibaura Institute of Technology Saitama Japan

**Keywords:** 11α,25‐dihydroxyvitamin D_3_ [11α,25(OH)_2_D_3_], CYP3A4(I301T), cytochrome P450, dependent rickets, gain‐of‐function mutation, type 3 rickets, vitamin D, vitamin D inactivation/C‐11 hydroxylation

## Abstract

Rickets, a disorder of bone formation, was originally known as nutritional rickets due to vitamin D deficiency. Advances in science have since identified various genetic forms, typically involving loss‐of‐function mutations in vitamin D activation or other mineral metabolism pathways. Recently, type 3 rickets was identified as a previously undescribed gain‐of‐function mutation in CYP3A4 (Ile301Thr). This mutant enzyme leverages the unique features of cytochrome P450 to produce an inactive vitamin D metabolite, 11α,25(OH)_2_D_3_, resulting in insufficient active vitamin D. The discovery of this unique gain‐of‐function aetiology and its associated metabolite opens a significant new direction in rickets research.

## Graphical abstract

Rickets, a bone disorder, was historically categorised into either nutritional (vitamin D deficiency) or genetic forms involving loss‐of‐function mutations in mineral metabolism. Recently, a new mechanism, type 3 rickets, was discovered to be caused by a gain‐of‐function mutation in CYP3A4 (Ile301Thr). This mutant enzyme generates a previously unknown inactive vitamin D metabolite, 11α,25(OH)_2_D_3_, leading to insufficient active vitamin D. The discovery of this unique gain‐of‐function aetiology and its associated metabolite opens a significant new direction in rickets research.

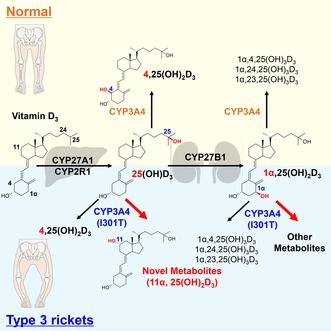

**Abbreviations**
11α,25(OH)_2_D_3_
: 11α,25‐dihydroxyvitamin D_3_
1α,25(OH)_2_D_3_
: 1α,25‐dihydroxyvitamin D_3_
1α,4,25(OH)_3_D_3_
: 1α,4,25‐trihydroxyvitamin D_3_
25(OH)D_3_
: 25‐hydroxyvitamin D_3_
4,25(OH)_2_D_3_
: 4,25‐dihydroxyvitamin D_3_
FGF23: fibroblast growth factor 23
PHEX: phosphate regulating endopeptidase homolog, X‐linked
PTH: parathyroid hormone
VDR: vitamin D receptor
VDRR: vitamin D‐resistant rickets
XLH: X‐linked hypophosphataemia

## Introduction

**Fig. 1:**
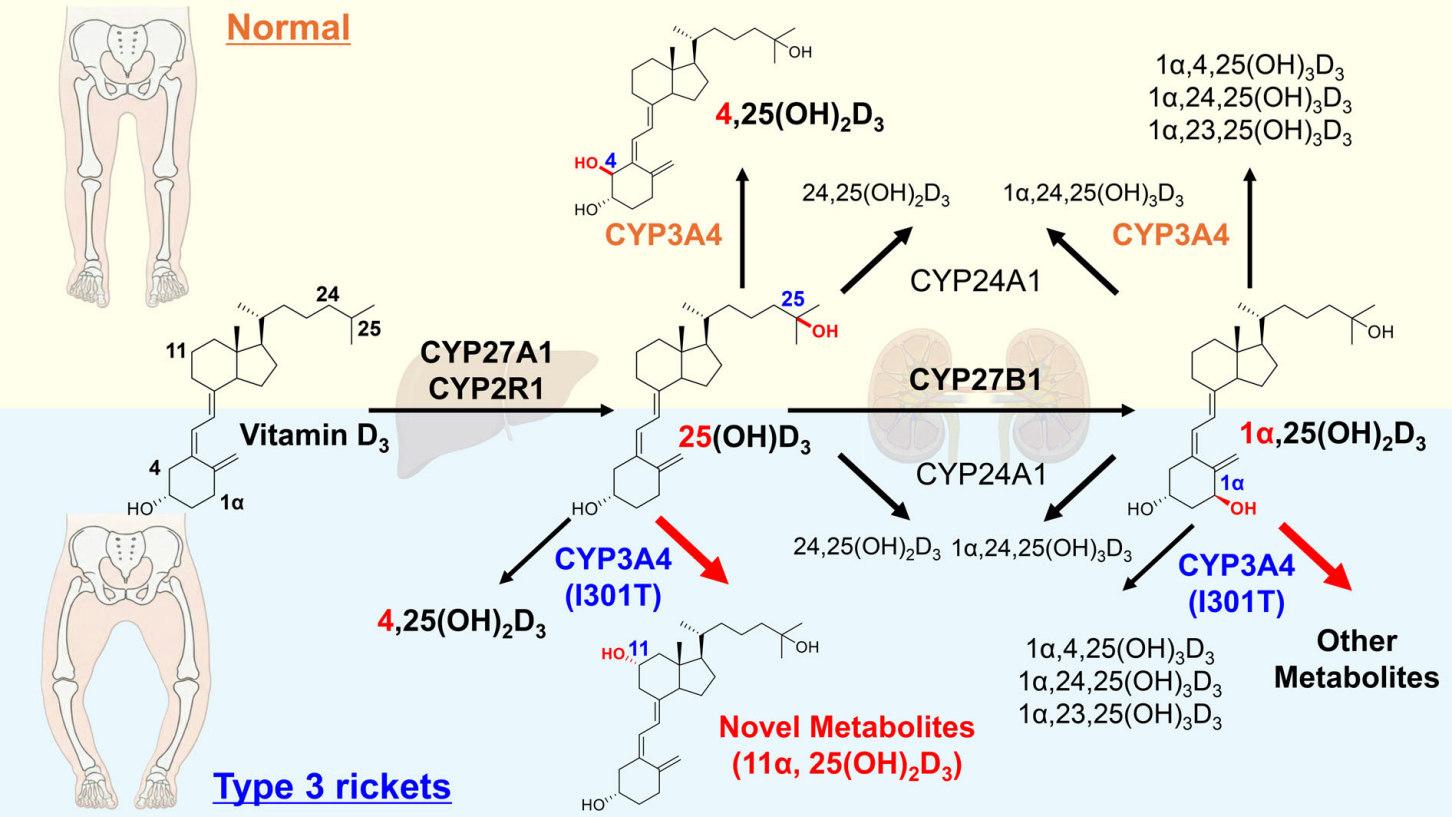
Vitamin D_3_ metabolism in normal individuals and in type 3 rickets caused by a gain‐of‐function mutation in CYP3A4 (I301T). The upper panel shows physiological vitamin D_3_ metabolism: conversion of vitamin D_3_ to 25(OH)D_3_ by CYP27A1/CYP2R1, then to 1α,25(OH)_2_D_3_ by CYP27B1, followed by inactivation via CYP24A1 and minor 4‐hydroxylation by wild‐type CYP3A4. The lower panel depicts the gain‐of‐function CYP3A4(I301T) variant, in which the mutant enzyme markedly enhances the conversion of 25(OH)D_3_ to 4,25(OH)₂D_3_ and to the newly identified 11α,25(OH)_2_D_3_. As a consequence, the conversion of 25(OH)D_3_ to 1α,25(OH)_2_D_3_ is profoundly reduced, leading to suppression of VDR‐mediated bone formation. These alterations reduce circulating active vitamin D_3_ metabolites and lead to the skeletal abnormalities characteristic of type 3 rickets.

Vitamin D (typically vitamin D_3_, or cholecalciferol) is a critical prohormone that regulates Ca^2+^ and phosphate concentrations in the body. Because its physiological actions extend beyond those of a conventional vitamin, it is generally regarded as a hormone‐like molecule. Vitamin D_3_ is synthesised from 7‐dehydrocholesterol (provitamin D_3_) in a two‐step process [1]. A photochemical reaction induced by sunlight (particularly UVB) first converts 7‐dehydrocholesterol into a 9,10‐secosteroid (previtamin D_3_), which is then converted to Vitamin D_3_ by thermal isomerisation. The resulting Vitamin D_3_ is subsequently hydroxylated in the liver to 25‐hydroxyvitamin D_3_ [25(OH)D_3_], and 25(OH)D_3_ is further hydroxylated in the kidney to 1α,25‐dihydroxyvitamin D_3_ [1α,25(OH)_2_D_3_], the biologically active form [1] (Fig. 1). This active metabolite binds to the vitamin D receptor (VDR) and activates gene transcription. To maintain mineral homeostasis, both 25(OH)D_3_ and 1α,25(OH)_2_D_3_ undergo further hydroxylation to inactive metabolites (Fig. 1). These reactions are catalysed by specific haem‐containing monooxygenases, namely cytochrome P450 enzymes such as CYP24A1. Because Ca^2+^ and phosphate levels require tight regulation, precise control of Vitamin D_3_ activity is essential for health, and defects in its activation or inactivation pathways can lead to severe disorders, most notably rickets.

## History of rickets research

Rickets is a disorder of bone formation, with descriptions dating back to the Greek and Roman eras, but it was not clearly recognised as a distinct clinical entity until the seventeenth century. It subsequently became a major public health problem in the United Kingdom, where it was notoriously referred to as the ‘English disease’. This period coincided with a marked increase in charcoal use and industrialisation, leading to severe air pollution. The resulting smog reduced UVB exposure and, as is now recognised, caused widespread Vitamin D_3_ deficiency. Nevertheless, the aetiology of rickets remained unclear until the late nineteenth century. In 1881, Barker reported that rickets was common in large cities but rare in rural areas [2], suggesting a link between sunlight and the disease. By the early twentieth century, it had been shown that UVB exposure could cure rickets [3], consistent with the fact that Vitamin D_3_ is synthesised in the skin upon UV irradiation. Vitamin D_3_ supplementation thus became the standard treatment, and this form of the disease is now classified as nutritional rickets.

Clinicians later identified cases of rickets that were refractory to Vitamin D_3_ supplementation. The first detailed study of this condition was conducted by Albright in the 1930s [4]. Because conventional treatment was ineffective, the disorder was termed vitamin D‐resistant rickets (VDRR), and Albright's work suggested a hereditary basis. It is now known that activation of vitamin D_3_ requires two‐step enzymatic hydroxylation by the cytochrome P450 isoenzymes CYP2R1 and CYP27B1 [1] (Fig. 1). Rickets caused by mutations in these enzymes is classified as vitamin D‐dependent rickets type 1 (VDDR1) [5]. Since activated Vitamin D_3_ exerts its effects by binding to the VDR [1], mutations in VDR itself give rise to type 2 rickets (VDDR2) [5]. These forms typically result from loss‐of‐function mutations. Beyond the vitamin D pathway, disorders involving parathyroid hormone (PTH) and fibroblast growth factor 23 (FGF23) also disturb mineral metabolism regulated by the PTH–vitamin D–FGF23 axis [6]. The rickets described by Albright, now known as X‐linked hypophosphatemia (XLH), belongs to this group. XLH is caused by a loss‐of‐function mutation in the Phosphate regulating endopeptidase homolog, X‐linked (*PHEX*) gene, resulting in impaired degradation of FGF23 and consequent FGF23 excess [7]. Thus, rickets is not merely a nutritional deficiency but can also arise from genetic defects within the complex PTH–vitamin D–FGF23 axis that regulates mineral homeostasis.

## Discovery of type 3 rickets and its molecular basis

In 2018, Roizen *et al*. reported a novel type of the disease, designated type 3 rickets, caused by a mutation in CYP3A4 [8]. Unlike types 1 and 2, which result from loss‐of‐function mutations, type 3 rickets is due to a gain‐of‐function Ile301Thr (I301T) mutation that selectively enhances CYP3A4 monooxygenase activity towards 25(OH)D_3_ without altering its other catalytic functions (Fig. 1). The activity of the CYP3A4(I301T) variant towards 25(OH)D_3_ is nearly double that of CYP24A1, the principal enzyme responsible for vitamin D_3_ metabolism. As a consequence of this accelerated catabolism, systemic 1α,25(OH)_2_D_3_ fails to reach concentrations sufficient for VDR activation. Whereas CYP24A1 hydroxylates vitamin D_3_ at the C‐24 position, Roizen *et al*. reported that CYP3A4(I301T) hydroxylates 25(OH)D_3_ at the C‐4 position, generating 4,25(OH)_2_D_3_ and 1α,4,25(OH)_3_D_3_ [8] (Fig. 1). In patients with type 3 rickets, the ratio of 4,25(OH)_2_D_3_ to 25(OH)D_3_ is markedly increased, suggesting that C‐4 hydroxylation represents an inactivation pathway. However, that initial study did not include detailed biochemical characterisation.

The paper by Nakaya *et al*. in this issue provides the first in‐depth biochemical analysis of type 3 rickets, elucidating its molecular mechanism [9]. The authors characterised the CYP3A4(I301T) variant and identified a novel Vitamin D_3_ metabolite underlying the disease phenotype. They showed that CYP3A4(I301T) predominantly hydroxylates the C‐11 position of 25(OH)D_3_, producing 11α,25(OH)_2_D_3_, which is formed in substantially greater amounts than 4,25(OH)_2_D_3_ (Fig. 1). Although 4,25(OH)_2_D_3_ was previously regarded as the key metabolite, more recent evidence indicates that it retains the capacity to bind to and activate VDR [10], implying that C‐4 hydroxylation does not necessarily terminate Vitamin D signalling. By contrast, Nakaya *et al*. demonstrated that 11α,25(OH)_2_D_3_ is unable to activate VDR. Their study thus provides the first biochemical identification of a genuinely inactive Vitamin D_3_ metabolite generated specifically by the CYP3A4(I301T) variant [9].

## Type 3 rickets and cytochrome P450 enzymes

As noted above, vitamin D_3_ hydroxylation is catalysed by cytochrome P450 monooxygenases, and both type 1 and type 3 rickets are therefore genetic disorders of P450 enzymes. They arise, however, from opposing mechanisms: loss‐of‐function in type 1 and gain‐of‐function in type 3. Cytochrome P450 enzymes constitute a major superfamily. In humans, 57 isoenzymes are generally classified into two groups [11]. The first group comprises enzymes with narrow substrate specificity that participate in the synthesis and turnover of endobiotics, such as steroid hormones and vitamin D. The second group consists of broadly specific enzymes that are typically induced by xenobiotics or endogenous metabolites. CYP3A4 is a prominent member of the second group and is responsible for metabolising more than 50% of clinically used drugs [12].

CYP3A4 is notable for its ability to oxidise substrates ranging from small molecules (molecular weight < 500) to large macrocycles (molecular weight > 1000). Kinetic studies have shown that CYP3A4 displays non‐Michaelis–Menten behaviour, consistent with its capacity to accommodate multiple substrates simultaneously [13]. Crystallographic studies [14] have clarified the structural basis of this promiscuity: a large, malleable active‐site cavity permits substantial conformational plasticity [15].

Nakaya *et al*. propose that the I301T mutation confers novel catalytic activity through a subtle structural change [9]. Although crystallographic confirmation is still lacking, their structural model provides a coherent explanation. In the wild‐type enzyme, the Ile301 side chain contains a δ‐carbon that sterically hinders 25(OH)D_3_ from adopting a binding orientation compatible with C‐11 hydroxylation. Substitution with Thr removes this steric constraint and allows 25(OH)D_3_ to bind via a hydrogen bond to Thr301. This alternative binding mode positions the C‐11 atom for hydroxylation, thereby generating the inactive metabolite.

## Concluding remarks

The study by Nakaya *et al*. elucidates the molecular mechanism of type 3 rickets through rigorous biochemical analysis [9]. Their structural model indicates that replacement of a single bulky hydrophobic residue (Ile) with a polar residue (Thr) can markedly alter substrate orientation and specificity. In addition, given that C‐4‐hydroxylated vitamin D_3_ retains biological activity, the identification of C‐11 hydroxylation as the inactivation pathway resolves an outstanding inconsistency in the field (Fig. 1). Intracellular vitamin D_3_ metabolism has been the subject of numerous investigations. In the present study, a gain‐of‐function mutation in CYP3A4 has revealed a previously unrecognised pathway of vitamin D metabolism and identified a novel vitamin D metabolite (e.g. 11α,25(OH)_2_D_3_). Functional alterations in vitamin D–metabolising enzymes have now been shown to reshape the profile of vitamin D metabolites in ways that contribute to disease. The future identification of novel vitamin D metabolites would open new avenues for understanding vitamin D‐related disorders and developing therapeutic strategies.

## Conflict of interest

The authors declare no conflict of interest.

## Author contributions

TS contributed to the conception of the work and to the drafting and critical revision of the commentary. YH contributed to the drafting and critical revision of the commentary and prepared the figure.
